# Comparing of letrozole versus clomiphene citrate combined with gonadotropins in intrauterine insemination cycles

**Published:** 2012-01

**Authors:** Soheila Akbari, Maryam Ayazi Roozbahani, Fatemeh Ayazi Roozbahani

**Affiliations:** 1 Department of Obstetrics and Gynecology, Asalian Hospital, Lorestan University of Medical Sciences, Khoramabad, Iran.; 2 Department of Obstetrics and Gynecology, Shahid Beheshti University, Clinical Center for Infertility of Erfan, Tehran, Iran.; 3 Department of Microbiology, Lavasani Hospital, Shahid Beheshti University, Tehran, Iran.

**Keywords:** *Letrozole*, *Clomiphene citrate*, *Gonadotropin*, *IUI*

## Abstract

**Background:** Clomiphene citrate (CC) an agonist and antagonist of estrogen, is the first line treatment in ovarian stimulation. Anti-estrogenic effect of CC in endometrial thickness and cervical mucus has negative effect on pregnancy rate. Letrozole is an Aromatase Inhibitor has been seen that has acceptable pregnancy rate compared to CC.

**Objective**: The aim of this study was to compare the efficacy of letrozole and clomiphene citrate (CC) with gonadotropins for ovarian stimulation in women candidate for intrauterine insemination (IUI).

**Materials and Methods:** One hundred sixty patients eligible to IUI therapy enrolled in this study. Patients randomized to two groups: group A (received letrozole-gonadotropin) and group B (received CC-gonadotropin). In group A (n=80) letrozole was given on days 3-7 of the menstrual cycles. In group B clomiphen citrate was given like letrozole combined with human menopausal gonadotropin (hMG) administered every day starting on day 8. Ovulation was triggered with urinary HCG when the leading follicle (s) reached 18 mm in diameter. A single IUI was performed 36-40 hours later. The ovarian stimulation response (E_2_ levels and number of follicles, clinical pregnancy and endometrial thickness) was primary outcome.

**Results:** Both groups were similar in demographic characteristics. There was a significantly lower peak serum E_2_ level in the letrozole group compared with CC. (236±86 Vs. 283±106 pg/mL, respectively; p<0.002). The number of mature (>18 mm) preovulatory follicles was significantly higher in CC group than letrozole group (2.2±.68 Vs. 2.02±0.63 respectively; p=0.025). Endometrial thickness measured at the time of hCG administration was significantly higher in letrozole group. (9.08±1.2 mm Vs. 8.1±1.9 mm; p=0.0001). The clinical pregnancy rate was comparable between two groups.

**Conclusion** **:** Letrozole is a good and cost-effective alternative to CC in IUI cycles.

## Introduction

Clomiphene citrate (CC) is the first line treatment in ovulatory dysfunction and infertility patients. Clomiphene citrate is an agonist and antagonist of estrogen, generally acting as a competitive estrogen antagonist at physiological female estrogen levels (1, 2). 

Clomiphene citrate is able to stimulate ovulation by competing with estrogen for binding to the hypothalamic estrogen receptors. It can induce ovulation in 73-87% of patients but pregnancy rate is lower (10-20% per cycle) (3, 4). 

This disparity seems to be due to the anti-estrogenic mechanism of action of CC which involves lasting estrogen receptor (ER) depletion (5) and may have a negative effect on the quality and quantity of cervical mucus and endometrial development, which may cause implantation failure, luteal phase defects and significant thinning of the endometrium, which is dose dependent (6-8).

It was shown that 20-25% of the women are resistant to CC and fail to ovulate (3). Studies have shown a significant difference between rate of ovulation and pregnancy and a higher abortion rate in patients undergoing CC therapy. In such cases, other option is: adding gonadotropins, though it is associated with an enhanced risk of multiple pregnancies and ovarian hyper stimulation. 

Gonadotropins (FSH/HMG) used in combination with CC decrease the dose required for optimum stimulation and make it more cost-effective in women who fail to respond to CC treatment. Recently, aromatose inhibitors (AIS) have extensively been used in inducing ovulation in anovulatory and ovulatory infertile women with inadequate response to CC. 

Letrozole, a third-generation aromatase inhibitor (AI), inhibits estrogen production and this; in turn increase state GnRH release and pituitary follicle-stimulating hormone (FSH) synthesis. Letrozole, a highly selective AI, in several studies has been investigated and it was confirmed that letrozole is associated with acceptable pregnancy rates, cost-effectiveness, decreased side-effects and patient convenience, as compared to gonadotropins (5-9). 

Some prospective pilot studies have been performed and the results showed that letrozole cycles have a significantly higher pregnancy rate than CC in gonadotropin-combined IUI cycles. These researches believed that their favorable outcomes could be attributed to a thicker endometrium and a lower level of E_2_ (5, 10). 

In addition it was shown that the use of letrozole-combined gonadotropins versus CC-gonadotropin in IUI cycles had a significantly lower serum E_2_ level on the day of hCG and the number of mature follicles. However, the same results were found in terms of pregnancy rate and endometrial thickness on the day of hCG administration between the two groups (10). 

Therefor we did this randomized clinical trial to evaluate the clinical outcomes of letrozole and CC in gonadotropin-combined IUI cycles.

## Materials and methods

**Patients’ selection**

This randomized single-blinded prospective controlled clinical trial was conducted in a tertiary infertility care unit, Kosar Infertility Center and a university hospital, in Tehran, Iran.

This study was approved by the ethics committee of Lorestan Medical University. In total, 160 women, younger than 40 years of age, with patent fallopian tubes and infertility of more than 1 year, who were candidate for IUI and gonadotropin therapy, were included in this study. Patients with a history of liver and kidney failure, cardiovascular disease and diabetes were excluded from the study. These women were randomly assigned into two different ovarian stimulation protocols categorized as group A and B.

The patients in the letrozole group (Group A) received 5 mg letrozole for 5 days (Femara, Novartis, Quebec, Canada) from day 3 to 7 of menstrual cycle. In the CC-gonadotropin group (Group B), CC 100 mg was given for 5 days starting from day 3 of menstrual cycle. In addition, in both group human menopausal gonadotropin (hMG, Pergonal, Serono) 150 IU was administered every day, starting on day 8 until human chorionic gonadotropin (hCG) administration. 

Response to treatment was assessed by performing vaginal ultrasound to check the number, size of follicles and endometrial thickness. Sonography was done every other day from day 10 of the cycle by a single radiologist. When mature leading follicle(s) reached >18^o^mm in diameter, urinary hCG (Profasi, Serono, Italy) in a dose of 10000IU was given and IUI was performed 36-40 hours later.

The main outcome measurement was clinical pregnancy rate. Clinical pregnancy was defined when an intrauterine gestational sac was visible by ultrasonography. Our secondary outcome measurements were the number of mature follicles (18 mm or more in diameter), serum level of E_2_ and endometrial thickness measured on the day of hCG administration and abortion rates )loss of pregnancy before 20^th^ week of gestation). 

**Statistical analysis**

SPSS version 12.0 software was used for statistical analyzing of data. The differences in the study and control group were analyzed using chi-squared and t-test. P-value less than 0.05 was considered statistically significant.

## Results

The basal characteristics of both groups are shown in table I. There was no significant difference between group A and B with respect to age and duration of infertility. There was a significantly lower peak serum E_2_ level in the group receiving letrozole compared with CC (236±86 Vs. 283±106 pg/mL, respectively; p<0.002) and the number of mature (>18 mm) pre-ovulatory follicles was significantly higher in CC compared with letrozole group (2.2±0.68 Vs. 2.02±.63 respectively; p=0.025). 

A significantly higher endometrial thickness was observed at the time of hCG administration in patients that received letrozole (9.08±1.2 mm Vs. 8.1±1.9 mm; p=0.0001) The clinical pregnancy rate was not significantly higher in letrozol group compared with CC group (17 patients (21.3%) Vs. 11 patients (13.8%), respectively). 

Full-term pregnancies were not significantly higher in Two cases of abortion were observed in the clomiphene citrate group in the first trimester, whereas none the letrozole group than the clomiphene group: 17 (21.3%) Vs. 9 (11.3%) p=0.146. There was no fetal anomaly in both groups. 

Fetal anomaly was defined as any structural anomaly and detected by ultrasound performed in first trimester and at 16-18 weeks.

**Table I:** Basic characteristic and different variables in the letrozol and clomiphene citrate groups base on mean ±SD

**Variable**	**Group A** **(Letrozole group n=80)**	**Group B** **(Clomiphene group n=80)**	**p-value**
Age (years)	28.50 ± 4.7	28.40 ± 4.5	NS
Duration of infertility (years)	6.3 ± 3.9	5.05 ± 2.4	NS
Endometrial thickness on day of HCG administration (mm)	9.08 ± 1.2	8.1 ± 1.9	0.0001
No. of mature follicle	2.02 ±.63	2.2 ± 0.68	0.025
E_2_ level on day of HCG administration (pm/ml)	236 ± 86	283 ± 106	0.002

**Table II:** Clinical outcomes in letrozole and clomiphene citrate group.

**Variable**	**Group A (Letrozol group n=80)**	**Group B (Clomiphene group n=80)**	**p-value**
Clinical` pregnancy	17 (21.3 %)	11 (13.8%)	NS (0.146)
Miscarriage	0 (0%)	2 (2.5 %)	NS (0.454)
Full term pregnancy	17 (21.3 %)	9 (11.3 %)	NS (0.062)

## Discussion

The results of this study showed similar pregnancy and fewer abortion rates in letrozole group compared with clomiphene in IUI cycles. There were no significant differences in the duration of infertility and age in both groups. 

Mean endometrial thickness on the day of HCG administration was significantly less in clomiphene citrate group than those who received letrozole (9.08±1.2 mm vs. 8.1±1.9 mm; p=0.0001) that is similar to results of Barroso *et al *(2006) (11). However, in the studies performed by Al-Fozan *et al* (2004), Jee *et al *(2006), and Davar *et al* (2006) they did not find any significant relationship between these two groups (12,13,16,18). 

This lower endometrial thickness in CC groups is attributed to the anti-estrogenic effect of CC in endometrial and cervical mucosa led to lower pregnancy rate as compared to letrozole, although this was not significant (17 patients (21.3%) Vs. 11 patients (13.8%), respectively). That is similar to results of Barroso (2006), and Jee BC (2006) (11, 13). To correct the negative impact of CC, the next step could be the induction of ovulation with gonadotropins which increase both the cost and risk associated with treatment. 

In our study, one patient in CC group and no patient in letrozole group had twin pregnancy (the difference not being significant. In addition, two patients in CC group had abortion, but in letrozole group we had no abortion, 2 (2.5%) vs. 0 (0%) respectively that was similar to results of Jee BC (2006), but in the study of Al-Fozan (2004) and Davar *et al* (2006) they found higher miscarriage rate in CC group (12, 13, 17).

"High supra physiological levels of estrogen attained during ovarian stimulation with clomiphene citrate may explain some of the adverse effects of clomiphene on the outcome of infertility treatment, although reducing estrogen synthesis by aromatase inhibitor may ameliorate such deleterious effects" (14). 

In some articles has been showed that "aromatase inhibitors can prevent peripheral estrogen production in patients in whom peripheral estrogen secretion is increased "(Bast *et al* 2000). The use of aromatase inhibitors in the initial follicular phase has a negative feedback effect on the hypothalamus and pituitary glands, thereby causing GnRH, LH and FSH secretion with result on ovarian follicular growth stimulation. "They may also have direct action on the ovaries and increase follicular sensitivity to FSH. High exogenous FSH or low estrogen production because of aromatase inhibitors will lead to growth of one or more ovarian follicles" (Bast *et al* 2000), as we had no twin pregnancy in letrozole group compared to CC group (14, 15).

In conclusion, the results of this study showed that letrozole is a good alternative to clomiphene citrate, or it can be a first-line drug in ovarian stimulation and treatment of anovulation. Use of aromatose inhibitors, letrozole, can induce ovulation comparable to CC without any adverse effect on endometrium and with comparable pregnancy rate, and lower abortion rate compared with CC.

## Footnote

***Registration ID in IRCT: IRCT138712141744n1***
